# The PROUST hypothesis: the embodiment of olfactory cognition

**DOI:** 10.1007/s10071-022-01734-1

**Published:** 2022-12-21

**Authors:** Lucia F. Jacobs

**Affiliations:** Department of Psychology, University of California, Berkeley, 2121 Berkeley Way, Berkeley, CA 94720-1650 USA

**Keywords:** Hippocampus, Locomotion, Olfaction, Respiration, Spatial cognition

## Abstract

The extension of cognition beyond the brain to the body and beyond the body to the environment is an area of debate in philosophy and the cognitive sciences. Yet, these debates largely overlook olfaction, a sensory modality used by most animals. Here, I use the philosopher’s framework to explore the implications of embodiment for olfactory cognition. The philosopher’s 4E framework comprises *embodied* cognition, emerging from a nervous system characterized by its interactions with its body. The necessity of action for perception adds *enacted* cognition. Cognition is further *embedded* in the sensory inputs of the individual and is *extended* beyond the individual to information stored in its physical and social environments. Further, embodiment must fulfill the criterion of mutual manipulability, where an agent’s cognitive state is involved in continual, reciprocal influences with its environment. Cognition cannot be understood divorced from evolutionary history, however, and I propose adding *evolved*, as a fifth term to the 4E framework. We must, therefore, begin at the beginning, with chemosensation, a sensory modality that underlies purposive behavior, from bacteria to humans. The PROUST hypothesis (*perceiving and reconstructing odor utility in space and time*) describers how olfaction, this ancient scaffold and common denominator of animal cognition, fulfills the criteria of embodied cognition. Olfactory cognition, with its near universal taxonomic distribution as well as the near absence of conscious representation in humans, may offer us the best sensorimotor system for the study of embodiment.

## Introduction

A critical question for the field of animal cognition is that of boundaries: what exactly is cognition and who has it? Studies of minimal cognition, e.g., cognition in plants, bacteria and other species lacking nervous systems, are already challenging the field (Duijn 2006). In philosophy and cognitive science, the definition of cognition is challenged even further by the concept of the embodied and extended mind (Clark and Chalmers 1998; Keijzer 2017; Varela et al. 1992). Here, a brain is only one part of a mind, which can exist embodied, embedded and extended, in its environment (Carter et al. 2018). Philosophers define embodied cognition as follows: “The properties of an organism’s body limit or constrain the concepts an organism can acquire.” (Shapiro and Spaulding 2021). Thus, at the very heart of embodied cognition is the concept that different bodies will necessarily shape different minds, a fundamental tenet of animal cognition.

The study of embodied cognition entertains a diversity of interpretations, each with a range of propositions from the modest to the radical. One such framework is “4E cognition”. Here embodiment is parcellated into four levels of analysis: cognition of the body (*embodied* cognition), cognition as related to the sensory inputs and physical affordances of the individual’s environment (*embedded* cognition), how an individual’s action creates its perceptions and concepts (*enacted* cognition) and finally, the information that an individual accesses that is stored in physical and social environments externally to its brain and body (*extended* cognition) (Shapiro and Spaulding 2021).

But animal cognition could add a fifth E, *evolved.* Excellent reviews have addressed the question of comparative embodied cognition, in cephalopods, domestic dogs and spiders (Cheng 2018; Japyassú and Laland 2017). But in the discussion of embodied cognition in both philosophy and comparative cognition, there is a notable omission: olfactory cognition.

This oversight is serious; indeed one could argue that it endangers the enterprise. Chemosensory perception and action is the common denominator of animal species, both aquatic and terrestrial, from single- to multicellular species (Ache and Young 2005; Bargmann 2006; Eisthen 1997; Papini 2008). It is also a primary sensory modality in minimally cognitive species, such as bacteria and plants. In prokaryotes, it is the most important sensory modality recruited for spatial orientation (Gelperin 2014; Parsek and Greenberg 2005). Spatial orientation to odors may arguably have been the class of associative learning that was the impetus for the radiation of animal phyla in the Cambrian (Ginsburg and Jablonka 2010; Jacobs 2012). It is even possible that selection for olfactory navigation in the genus *Homo* may have led to the evolution of the nasal pyramid, and hence shaped the evolution of our own species (Jacobs 2019).

For these reasons, any discussion of embodied cognition, even in humans, cannot neglect chemosensation. But understanding embodiment is also critical for animal cognition. Understanding mechanisms of cognition and behavior demands an understanding of evolution and adaptation (Cisek and Hayden 2022; Krakauer et al. 2017). The surest path to this goal is through structured comparisons of convergent and divergent adaptations across taxa (Arnold and Nunn 2010; Barton et al. 1995; MacLean et al. 2012). If the question we are trying to answer is the embodiment of cognition, the logical course of action is to build structured comparisons among diverse species. Ideally, such species would differ along specific parameters in embodiment (i.e., morphology), enactment (i.e., sensorimotor competency), embeddedness (i.e., sensory ecology) and extension (i.e., the structure of their cognitive niche). To do this effectively would require the widest possible sweep of taxonomic breadth. This, of course, could best be accomplished by studying convergence and divergence in chemosensory cognition, building upon already impressive work in comparative and behavioral neuroscience (Ache and Young 2005; Baker et al. 2018; Corey and Ache 2016; Eisthen 2002; Laurent 2002).

Finally, not only is olfaction central to embodied cognition, but it is possible that the whole enterprise will fail without it. This is because olfaction offers direct evidence for the radical claim of embodied cognition that the very concept of representation is misleading. Discussions almost exclusively centered on visual cognition (Carter et al. 2018) overlook decades of work in olfactory cognition that questions whether an odor is represented consciously at all (Herz and Engen 1996; Zucco 2007).

### Olfaction and representation

The neuroanatomy of the main olfactory system (hereafter olfactory system) may explain the unique attributes of olfactory cognition. The olfactory system, a primary component of the vertebrate brain Bauplan, is the only sensory system to bypass the thalamus, the relay station of the diencephalon (Striedter 2005). The thalamus mediates conscious attention in humans and is activated when the stimuli are visual or auditory stimuli, but not with olfactory stimuli (White 2012). For this reason, Kay and Sherman proposed that the main olfactory bulb (hereafter olfactory bulb) serves as its own relay station, sending inputs to the olfactory cortex, i.e., the piriform cortex. This structure also receives inputs from taste, visual, auditory and somatosensory systems. The olfactory bulb thus uniquely projects directly to this multi-sensory cortical structure (Kay and Sherman 2007).

This privileged neural circuity may explain why untrained human participants find the conscious recall of a remembered odor difficult or impossible to perform, compared to the accurate performance of the same task using visual stimuli (Herz and Engen 1996). The presentation of an odor even modulates object visibility, both the identity and the duration of a visual stimulus (Zhou et al. 2017). Thus, odors are not only difficult to recall or label consciously, they also distort inputs from other sensory modalities and even compete with language processing (Herz 2020, pp. 472–482).

In addition to the lack of thalamic modulation, the olfactory system has robust, reciprocal projections to the amygdala, a limbic structure that subserves emotional learning and memory. This neural architecture may explain why odors are inherently emotional (hedonic). For example, the memory of the odor of a stimulus, such as popcorn, is encoded with greater emotional valency than the visual appearance or sound associated with the same stimulus (Herz 2012, 2016; Herz and Cupchik 1995; Herz and Schooler 2002; Kontaris et al. 2020).

The absence of a thalamic projection, combined with the important projections to emotional circuits, may explain why verbal encoding of odors is highly inefficient, yet odor learning and memory are remarkably resistant to decay. In a classic study, the accuracy to identify an odor decreased significantly after a 30 s retention interval. But after 30 s, there was only a 3% decrease in accuracy of recall after subsequent delays of 3 days, 1 month and even 1 year (Engen and Ross 1973). Such studies fuel a serious, ongoing debate whether olfaction is in itself a separate memory system, one with no distinction between short- and long-term memory (Herz and Engen 1996).

One reason why such questions remain unanswered is that we still lack a standard model of odor perception. We even lack a fundamental model of the neural code by which the olfactory brain identifies an odor, as current data support two competing theories, the pattern model and the vibration model (Herz 2020, pp. 464–468). Yet, another question which has not been definitely answered is how the brain encodes the odors it has identified, whether as the elements in a mixture or the mixture as a single synthesis (Barwich 2019; Herz 2020, pp. 468–470; Wilson and Stevenson 2010). In short, the olfactory system is a sophisticated cognitive system that exhibits unusual characteristics, including the difficulty of conscious representation. Instead, the olfactory system may be a uniquely emotional and unconscious learning and memory system (Wilson and Stevenson 2003; Zucco 2003, 2007). It is small wonder that olfaction was shunned for centuries by philosophers modeling the human mind as conscious and rational (LeGuérer 2002) and is only now being taken up by a new generation of philosophers (Barwich 2019; Batty 2010).

### The utility of olfaction

Yet, the majority of research in olfactory cognition, whether in humans or other species, is designed to study how the brain identifies and assigns valency to an odor, what could be called *diagnostic olfaction*. But this downplays or ignores a critical function of olfactory cognition, its role in spatial orientation, i.e., *directional olfaction* (Jacobs 2019). This is an important distinction because many paradoxes of olfactory anatomy and psychophysics can only be explained in terms of directional olfaction functions (Jacobs 2012, 2022; Marin et al. 2021). In the *olfactory spatial hypothesis* (Jacobs 2012), I discuss how unique patterns of allometry and neuroplasticity in the vertebrate olfactory system play a role in directional olfaction. For example, the relative size of the olfactory system can be explained by a species’ ability to orient using odors. Hence it follows that directional olfaction could be a primary selective force acting on the evolution of the olfactory system (Jacobs 2012).

This insight arose from a prior consideration that distributed gradients, such as odor plumes, had been missing from models of hippocampal function, a limbic brain structure critical for spatial navigation. Françoise Schenk and I addressed this in the *parallel map theory* of navigation (Jacobs and Schenk 2003). It was the first model of the hippocampus to incorporate olfactory gradients as orientation cues. We also proposed that orientation to such *directional cues* was the ancestral function of hippocampal homologues (e.g., medial pallium and medial cortex) in vertebrates (Jacobs 2003). More recently, I have proposed that it was the evolution of air breathing in lobe-finned fish, and their subsequent move to land as the first tetrapods, that led to directional olfaction becoming a primary function of the olfactory system in terrestrial vertebrates (Jacobs 2022).

But the data on directional olfaction in mammals is surprisingly sparse, apparently because of the assumption that the olfactory system is not spatial, but only diagnostic. Even studies of spatial orientation in highly olfactory species (e.g., laboratory rat and mouse, Order Rodentia, Family Muridae, Subfamily Murinae) actively eliminate odors and odor plumes as orientation cues (Jacobs 2022). This is partly due to the technical challenge of controlling such stimuli, but as a result, we have a poor understanding of the relationship between olfaction, space and the hippocampus (Jacobs 2012, 2022), despite Françoise Schenk’s early work in this area (Lavenex and Schenk 1997, 1998). This is changing, however, with studies of hippocampal function that explicitly build on the parallel map theory (Hagena and Manahan-Vaughan 2011; Kemp and Manahan-Vaughan 2007a, b) and the olfactory spatial hypothesis (Dahmani et al. 2018; Jacobs et al. 2015; Zhang and Manahan-Vaughan 2015). The impact of the olfactory spatial hypothesis has now reached beyond the hippocampus, with the discovery that the piriform cortex actively encodes the spatial location of odors (Poo et al. 2021). In short, there is increasing evidence that a primary function of the olfactory system is spatial orientation (Jacobs 2022).

In contrast to mammals, there is a rich literature of orientation to odors in insects (Baker et al. 2018; Vickers 2000; Vickers et al. 2001; Willis et al. 2013) and birds (Gagliardo 2013; Wallraff 2005). In birds, trained homing pigeons, as well as wild sea gulls and migrating songbirds, orient more accurately over long distances when their olfactory system is intact and functioning (Thorup et al. 2007; Wikelski et al. 2015). Recent studies of hippocampal place cells in food-storing and non-food-storing songbirds have demonstrated remarkable homologies with hippocampal function in mammals (Payne et al. 2021).

Many of these results in birds are concordant with an interpretation of avian navigation based on the parallel map theory (Jacobs and Menzel 2014). A navigator that moves over larger distances, such as flying birds or flying insects, is able to orient to the pattern of larger stimuli. In the case of olfactory landscapes, these could be based on the association of cardinal directions to known locations. This could include orientation to localized concentrations of odors, as in the mosaic map model of Floriano Papi, or gradients of atmospheric odors, as in the olfactory navigation model of Hans Wallraff (Gagliardo 2013). Although we lack evidence for this in birds, based on studies of plume orientation in flying insects, a larger scale of stimulus distribution should facilitate the ability to sample and orient to odor plumes. Whether odors are concentrated locally or distributed in gradients, it follows that using odors to orient will be more useful for species that navigate over larger distances. This may be one reason why mapping of space using odors is influenced by scale and why olfactory inputs are critical for accurate long-distance homing in displaced birds (Jacobs and Menzel 2014).

### The PROUST hypothesis

In his famous passage in the first volume of the novel, “In Search of Lost Time”, Marcel Proust offers perhaps the finest description of the recall of a flavor memory (Proust 2002). Now known as a ‘Proustian memory’, the passage describes how the flavor of a specific cookie dipped in a specific tea sparked the recall of a childhood memory. The passage illustrates key characteristics of olfactory memory: how an odor first activates an emotion which then triggers the effortful reconstruction of a spatio-temporal memory. The precision of Proust’s observations in this passage have inspired the design of studies in olfactory cognition (Herz 2016; Herz et al. 2004; Jellinek 2004). Jellinek has deconstructed the passage into no fewer than 11 hypotheses about olfactory memory, many of which have been confirmed empirically (Jellinek 2004). Here are selections from Proust’s iconic passage:

But at the very instant when the mouthful of tea mixed with cake crumbs touched my palate, I quivered, attentive to the extraordinary thing that was happening inside me. (Proust 2002, p. 45).

Where could it have come to me from—this powerful joy? I sensed that it was connected to the taste of the tea and the cake, but that it went infinitely far beyond it, could not be of the same nature. Where did it come from? What did it mean? How could I grasp it? (Proust 2002, p. 45).

Seek? Not only that: create. It is face-to-face with something that does not yet exist and that only it can accomplish, then bring into its light.

And I begin asking myself again what it could be, this unknown state which brought with it no logical proof, but only the evidence of its felicity, its reality, and in whose presence the other states of consciousness faded away. I want to try to make it reappear. I return in my thoughts to the moment that I took the first spoonful of tea. I find the same state again, without any new clarity. I ask my mind to make another effort, to bring back once more the sensation that is slipping away. And, so that nothing may interrupt the thrust with which it will try to grasp it again, I clear away every obstacle, every foreign idea, I protect my ears and my attention from the noises in the next room. But feeling my mind grow tired without succeeding, I now compel it to accept the very distraction I was denying it, to think of something else, to recover its strength before a supreme attempt. Then for a second time I create an empty space before it, I confront it again with the still recent taste of that first mouthful, and I feel something quiver in me, shift, try to rise, something that seems to have been unanchored at a great depth; I do not know what it is, but it comes up slowly; I feel the resistance and I hear the murmur of the distances traversed. (Proust 2002, p. 46).

Ten times I must begin again, lean down toward it. And each time, the laziness that deters us from every difficult talk, every work of importance, has counseled me to leave it, to drink my tea and think only about my worries of today, my desires for tomorrow, upon which I may ruminate effortlessly.

And suddenly the memory appeared. That taste was the taste of the little piece of madeleine which on Sunday mornings at Combray (because that day I did not go out before it was time for Mass), when I went to say good morning to her in her bedroom, my aunt Léonie would give me after dipping it in her infusion of tea or lime blossom. The sight of the little madeleine had not reminded me of anything before I tasted it […]. (Proust 2002, p. 47).

The PROUST hypothesis (*perceiving and reconstructing odor utility in space and time*) evokes Proust’s insight that olfactory cognition can evoke the reconstruction of an experience in distant space and time. These cognitive mechanisms can only be understood by retracing their evolutionary history, as with the hippocampus (Jacobs and Schenk 2003), main olfactory system (Jacobs 2012) and vomeronasal system (Jacobs 2022). If complex cognition first emerged in highly chemosensory animals, then the answer to many questions about 4E cognition may lie in understanding how this plays out in the olfactory cognition of species today.

### Defining the boundaries

To recapitulate the 4E framework (Shapiro and Spaulding 2021): the *embodiment* of cognition is its constraint by the morphology and competencies of the body. The *embedding* of cognition is how the physical environment in which that body is located shapes cognitive load; the more appropriate the environment, the smaller the cognitive load. The next level is *enactivism*, where cognition emerges as the mutual interactions of a sensorimotor system with its physical and social environments. Finally, these actions, which are both embedded and embodied, change and shape the *extended* social and physical environment, as “… the environmental and social resources that enhance the cognitive capacities of an agent are in fact *constituents* of a larger cognitive system, rather than merely useful tools for a cognitive system that retains its traditional location wholly within an agent’s nervous system….” (emphasis in the original) (Shapiro and Spaulding 2021, Sect. 2.3).

Of course, the problem with such a framework is “cognitive bloat”, where everything and hence nothing is cognition (Kaplan 2012). An accepted solution to this has been the concept of “mutual manipulability” (Craver 2009; Kaplan 2012). As defined in comparative cognition, “systematic manipulations of the object must affect the animal’s cognition, and changes in the animal’s cognition must affect the object, via some causal chain. Only when this two-way flow has been established can the object be considered part of the animal’s extended cognition.”(Cheng 2018, p. 6).

One such example is the analysis of orb-weaving in spiders as *extended cognition* (here used to represent all levels of embodied cognition) (Japyassú and Laland 2017). As a spider tightens the threads of a web, this creates a new environment where smaller insect prey can be detected. This change in prey detection feeds back on the attentional system of the spider. Thus, the spider’s cognition (attention and perception) is extended into its environment, i.e., its web (Cheng 2018; Japyassú and Laland 2017).

Cheng explains the mutual manipulability criterion more broadly as follows: “In general, information-seeking behavior that supports a cognitive enterprise often satisfies the mutual manipulability criterion. Kaplan (2012) gave the example of saccadic eye movements in humans to look repeatedly at a target to support working memory. To satisfy the mutual manipulability criterion, certain cognitive states must cause more or different kinds of information seeking, and the information seeking must help the enterprise.” (Cheng 2018, p. 11). He describes the example of a specific movement (a pirouette) made by a navigating ant when it reaches an ambiguous choice point. The pirouette does not enhance locomotion but instead functions to gather additional information. Thus, like the saccade, the ambiguity of the location (a cognitive state) leads to the information seeking of an action (the pirouette), which then changes the cognitive state and thereby satisfies the mutual manipulability criterion.

Yet, once again, these examples from ants and humans are derived from visual cognition. My goal here, given the importance of chemosensation in animal behavior, is to explore what we can learn by placing olfactory cognition in the 4E framework and adding *evolved* as a fifth level of analysis.

### Embodied cognition

As in the examples from ant and human vision, the act of an olfactory sample satisfies the mutual manipulability criterion, as a cognitive state that causes useful information seeking. In air-breathing terrestrial vertebrates using nasal respiration, this sample is embodied in the sniff. Air is inhaled through the nostril and moves through the nasal cavity to reach the olfactory epithelium, where odorants contact the olfactory receptors. The inspired air then continues through the pharynx, carrying oxygen to the lungs (Mainland and Sobel 2006). There are two forms of sampling in mammals and this is called orthonasal olfaction—inhalation through the nose. The other form is retronasal olfaction in mammals, where odors in the mouth are carried via expiration to the olfactory epithelium before being exhaled (Ni et al. 2015; Small et al. 2005).

An orthonasal sniff has two functions, olfaction and respiration, and thus respiration is an integral component of olfactory cognition (Mainland and Sobel 2006). As in vision, the sniff itself is necessary and sufficient to activate the olfactory brain in mammals. If the eye is kept motionless, a visual image is not detected, despite the photons hitting the retina. In human olfaction, if the odorant is experimentally placed in contact with the epithelial olfactory receptors but in the absence of a sniff, the odorant is also not perceived. Yet, a sniff, even in the absence of an odorant, activates the oscillation frequencies (e.g., theta) that are normally associated with odors during sniffing (Mainland and Sobel 2006).

Humans modulate their sniff when asked to imagine odors, increasing the volume of the sniff for pleasant odors and decreasing the volume for unpleasant odors (Mainland and Sobel 2006). There is also a ‘dialogue’ between cortex and olfactory bulb, that has been well documented in laboratory murines. When a laboratory rat is preparing to enact a sniff to earn a reward, its entorhinal cortex (a higher sensory integration structure that funnels information to the hippocampus), activates before the sniff and hence before the activation of the olfactory bulb. This top-down modulation of olfaction is driven by an expectation based on the rat’s prior learning experiences (Kay and Freeman 1998). There is also a ‘language’ for this dialogue, in the form of oscillatory dynamics, which are convergent in form and function between mammals and insects (Ache and Young 2005; Kay 2015; Laurent 2002).

The importance of the sniff for cognition goes beyond olfactory tasks: orthonasal respiration is not only necessary for olfactory perception but also for the consolidation of learning. It has been recently demonstrated that nasal respiration synchronizes disparate brain regions, enhancing the consolidation of learning, even in non-olfactory structures and in non-olfactory tasks (Heck et al. 2017, 2019; Sheriff et al. 2021; Tort et al. 2021). This effect has been demonstrated in humans: respiration through the nose, but not the mouth, facilitates memory consolidation (Arshamian et al. 2018). In laboratory mice, these appear to be top-down influences on nasal respiration (not vice versa) and occur even during REM sleep (Tort et al. 2021). I have proposed that these effects can be explained by the evolutionary history of air breathing in vertebrates (Jacobs 2022).

The act of odor sampling is also highly purposeful and dynamic. A sniff is ‘focused’ by changing its duration, intensity, volume and temporal pattern (Schoenfeld and Cleland 2006). The sniff is therefore active information seeking, not simply respiration (Jacobs 2019), and its structure is fine-tuned for this purpose, as in a visual saccade. This behavioral modulation of sniffing focuses the information seeking via the specialized structure of the inner nasal skeleton, which varies significantly among mammalian species (Valkenburgh et al. 2014; Zwicker et al. 2018). The focusing of the sniff further exploits the chromatographic function of the nasal epithelium, a function first proposed by Mozell and Jagodowicz (1973). The chromatograph organization of the epithelium was proposed to emerge due to the molecular properties of an odorant, which cause it to be absorbed in different zones along the olfactory epithelium; this later led to the theory of zonation (Schoenfeld and Cleland 2006). Both the chromatograph and zonation hypotheses have received empirical support. As predicted, laboratory rats adapt their sniff characteristics to the chemical structure of the odorant they are sniffing (Rojas-Líbano and Kay 2012).

This fine control of sniffing also modulates the perceived difference between the target odor and the background odor. Laboratory rats increase this difference by increasing the frequency of sniffing, thus creating an adaptive filter (Verhagen et al. 2007). Similarly, trained search dogs increase the frequency of their sniffing at decision points, at locations where they must make finer odor discriminations (Thesen et al. 1993).

The speed of the sniff also changes the spatial location of the samples being collected. In physics, the faster a fluid is transported through a tube, the greater is the area from which the sample is collected. The catchment areas are separated even farther if the tube is lengthened (True and Crimaldi 2017). Thus, rapid sniffing through two external nostrils should theoretically increase the spatial separation of the odor samples, thus enhancing stereo olfaction (Jacobs 2019). Stereo olfaction has been demonstrated to increase the accuracy of directional olfaction, both empirically (Catania 2013; Martin 1965; Wu et al. 2020) and in a computational analysis of real world odor plumes (Boie et al. 2018). Increasing the spatial separation between the input locations enhances directional olfaction even farther: in sharks, the further apart in space and thus in time that two odor samples are collected, the greater accuracy of determining the direction of the odor source (Gardiner and Atema 2010). The physical properties of how fluids move through tubes could explain the evolution of the mammalian external nose and why tube-shaped nostrils are found in certain olfactory navigating species in birds (e.g., tube-nosed seabirds, Order Procellariiformes) and mammals, including the evolution of the external nose in the genus *Homo* (Jacobs 2019).

Finally, a sniff, like a visual saccade, fulfills the mutual manipulability criterion, because the new information changes the cognitive state. But unlike vision, the act of sampling odors also disturbs the stimuli that are being sampled. The movement of sampling (sniffing, movement of antenna or other antenniform structures such as tentacles or antennules, movement of the head, casting of the body and other forms of locomotion) distorts the fluid dynamics of the air and hence the geometry of the plume. In addition, when an odorant is sampled, it is absorbed into the olfactory epithelium. Molecules that are not absorbed by the olfactory epithelium are mostly captured in the mucosal lining of the inner nose. In the bullfrog, this lining absorbed 78% of odor molecules that were not absorbed by the olfactory epithelium (Hornung and Mozell 1977). The act of olfactory sampling thus permanently removes a stimulus from the surrounding environment. The physical movements of sampling are no doubt more significant than the removal of odorants from the transport vehicle (air or water). Yet, from first principles, it is nonetheless possible that this unique aspect of olfaction, compared to vision or audition, could be important for modeling its embodiment.

Olfactory cognition thus demands intricate and purposeful sampling, with high dimensional dynamic sniffing, adapted to focus the deposition of odorant molecules in specific zones within an aerodynamic nasal environment. Olfactory sampling clearly fulfills the mutual manipulability criterion: the cognitive state leads to a movement which changes the environment, and this changed state in turn changes the cognitive state of the agent. In summary, from the morphology of the nose to the mode of respiration, olfactory cognition is embodied in structures outside the brain.

### Embedded cognition

Because of its unique properties, olfactory cognition is also deeply embedded in the physical affordances of the external odor landscape. Any significant movement of an odorant, whether in air or water, occurs not by diffusion, which is too slow to be biologically useful to an animal orienting in space, but by advection, transportation in a fluid (Koehl et al. 2001; Moore and Crimaldi 2004). Directional olfaction thus demands an integration of chemosensory and other embodied inputs to measure the movement of the transport vehicle, such as its speed or turbulence. Combined, these inputs allow the navigator to construct a movement strategy that allows it to orient even within a turbulent odor plume (Baker et al. 2018; Weissburg 2011).

This necessitates estimating hydrodynamic forces by aquatic species and aerodynamic forces in terrestrial species. Plumes differ in air and water, which constrains how odors can be sampled, e.g., the antennule flick of a crustacean (Koehl et al. 2001), versus the olfactory organ sampling of a fish. Extremely large odorants can be transported in water and useful to an aquatic species (fish can smell peptides using their olfactory system) but the chemicals must dissolve in water (Kishida 2021). This adds a different constraint on the makeup of an odor landscape for an aquatic animal. In contrast, odorants borne in air must be volatile, with small molecular weights. The affordance of a light volatile can be spatial orientation: the long-range detection of odors associated with a cardinal direction after long-distance spatial displacement, as in homing pigeons (Gagliardo 2013). House mice can safely identify the species and gender of an unknown mouse at a distance by sampling volatile odors, but need to have direct contact to identify the individual (Hurst and Beynon 2004). Thus, how odors are embedded in the physical environment constrains the kind of information that can be extracted from them.

This embedding of olfactory cognition means that the olfactory agent must have mechanisms in place to decode the movement of fluids (Baker et al. 2018; Vickers et al. 2001; Weissburg and Zimmer-Faust 1994), whether air (anemosensory) or water (hydrodynamics). Terrestrial mammals accomplish this ‘anemo-chemo cognition’ by integrating respiration, olfaction and aerodynamics in remarkable synchrony. In the laboratory murines, there is an orofacial system of neural control for dynamic sniffing. This synchronizes the sniff (respiration and olfaction), hippocampal oscillations associated with spatial learning (at the theta frequency) and the whisking movement of the vibrissae (Kleinfeld et al. 2014). Laboratory rats can encode wind direction using their vibrissae (Yu et al. 2016), which suggests that this finely tuned orofacial system is adapted for directional olfaction.

A final factor that influences embedded olfaction is the geometry of the physical landscape. This also changes the information content of odors. A southern facing slope, for example, increases in temperature more rapidly than a northern facing slope (Conover 2007a). The atmosphere, shape and texture of the terrain also influences the movement of odors and hence should also influence the behavior and cognitive states of predators and prey. Prey animals should theoretically exploit the odor landscape using hot, dry locations as a temporary refuge from olfactory predators (Conover 2007b). The movements of individuals in such a landscape would in turn alter the distribution of their odors in the environment, which would in turn change the cognitive state of the individuals attempting to locate them, either predators or prey.

### Enacted cognition

Enactivism can be described as the hypothesis that as an agent acts, it creates a perception of its world. “The key point, then, is that the species brings forth and specifies its own domain of problems …this domain does not exist “out there” in an environment that acts as a landing pad for organisms that somehow drop or parachute into the world. Instead, living beings and their environments stand in relation to each other through mutual specification or codetermination.” (Varela et al. 1992, p. 198). Much of what has already been reviewed supports this in olfactory cognition: the necessity of the sniff for an odorant to be perceived, the focusing of the sniff to the molecular structure of the target odorant, creating an adaptive filter by changing sniff frequency or increasing stereo olfaction by casting and/or sniff frequency, and finally integrating these inputs with the fluid mechanics of air or water.

Real-world examples can be seen in the directional olfaction of trained search dogs. Search dogs increase sniff rate at choice points, where they must make fine diagnostic decisions to determine the direction of travel of the human whose footprints they are tracking. Once the direction has been diagnosed, sniff frequency declines and speed of movement increases (Thesen et al. 1993). Dogs can determine the direction of travel by sampling five or more successive human footprints. The interpretation is that the complex odor mixture of human scent is a mixture of small and large molecules. Light volatiles disperse sooner after deposition of the mixture and hence their presence is evidence that the mark was made more recently in time (Alberts 1992; Baeckens et al. 2017; Scordato et al. 2007). A footprint that still retains light volatiles must have been made more recently than a footprint with a lower concentration of such molecules, and must indicate the most recent sample and hence the human’s direction of travel (Hepper and Wells 2005; Wells and Hepper 2003). Thus, diagnostic olfaction is enhanced to solve a problem in directional olfaction.

Since olfactory cognition depends so directly on the physics of the transport vehicle, meteorological conditions influence the detectability of air-borne odors. Olfactory discrimination is also impacted by atmospheric conditions; detection thresholds are higher in hotter, drier climates. Search dogs adapt their search strategy under such conditions. Dogs following a trail of human footprints under hot and dry conditions were less accurate and increased their sampling of the ground versus the air. This slowed their forward rate of progress, compared to a dog with its head up, who can run and sniff at the same time. When the signal is lost, the dog must resort to slower sampling of the heavier molecules that persist on the substrate, when the lighter molecules, which are easier to track but more likely to degrade in hot or windy conditions, can no longer be reliably followed (Jinn et al. 2020).

Similar issues must be faced by aquatic species tracking the hydrodynamics of odor plumes underwater, as has been studied in detail in crustaceans (Weissburg and Zimmer-Faust 1994); understanding the cognition underlying odor tracking in aquatic vertebrates such as fish would greatly increase our understanding of such enacted cognition. Seals, like rats tracking wind plumes with their vibrissae, track the hydrodynamic plumes of their prey with input from their vibrissae, although as secondarily aquatic mammals, seals have lost the ability to detect odors dissolved in water (Adachi et al. 2022; Dehnhardt et al. 2001; Kishida 2021). In summary, anemo-chemo cognition in air-breathing terrestrial animals supplies important examples of enacted olfactory cognition.

### Extended cognition

The classic example of the extended mind in humans is a notebook, allowing the inclusion of information that is stored in the environment, instead of the nervous system, and is easily accessible (Clark and Chalmers 1998). Scent marks are depositions of sociochemical odor mixtures on substrates such as the ground surface or vegetation. These and other sociochemicals are rich repositories of information, transmitting species, sex, age and individual identity, but also changes in state over time, including reproductive, disease, stress and even nutritional states (Kavaliers et al. 2020; Zala et al. 2004). Since these odors are repositories of information stored in the environment, they also satisfy the criterion of accessibility. As in a notebook, an individual may rely on information that is accessible in the environment (Clark and Chalmers 1998). Sociochemicals thus effectively act as public records of information, not dissimilar to the written language of humans.

The information is structured as a form of olfactory ‘social media’, a present and past record of social encounters, acted out in public. The meaning of a mark is determined by three of its characteristics: who, where and when. Just as the meaning of a human footprint to a search dog is dictated by its location in space and time, the same is true of scent marks (Hurst and Beynon 2004). To extract the full meaning of a mark, an agent will study spatial and temporal changes in the placement and composition of odor mixtures, over periods of minutes, hours or longer (Gosling and Roberts 2001). Since scent marks undergo predictable changes in composition, providing a unique time-stamping function, these changes can convey the competitive status of the individual that left the mark (Alberts 1992).

The control of this process is important. The main urinary protein found in the urine of male house mice functions not only as a marker of individual identity but is also structured to slow the degradation of the odor mixture. By increasing the longevity of a fresh signal, these expensive metabolites enhance a male’s competitive ability (Hurst et al. 1998). The spatial location is equally critical for determining the meaning of a scent mark. A small displacement between house mouse scent marks conveys important information about social competition to a conspecific (Hurst and Beynon 2004).

The dynamics of counter-marking—where a competitor places a new scent mark over or adjacent to a prior mark—adds the further dimension of a public competition, witnessed by all. The age and location of a male’s countermark gives observers time-stamped evidence that a social competition between known antagonists has occurred. The geometry of countermarks (which mark is uppermost, which is broken) is a further source of information (Johnston 2003; Johnston et al. 1995; Tomlinson and Johnston 1991). This record of social competition is public, long lasting and accessible to anyone tracking the competitive ability and history of the contestants (Hurst and Beynon 2004). A mark placed in a particular time and space can convey ownership or a challenge, demonstrating that a non-territory holder has invaded and marked the property of another. A scent mark can directly advertise physiological state (Kavaliers et al. 2020; Wyatt 2010) or indirectly, that the signaler has an energetic budget sufficient to visit and mark widely dispersed locations (Gosling and McKay 1990). Finally, another source of public information is odors that an individual carries with them, on their bodies. Complex sociochemicals may be emitted during social interactions, such as displays of scent glands and ritual urination (Alberts 1992; Drea 2015). Thus, as extended sources of information, sociochemicals exist in the physical and social domains, but can occur either separate or coincident in time and space from a direct social encounter.

Finally, the environment holds a wealth of information about odor utility in space and time, that extends beyond a species’ sociochemicals. Any state-dependent odor could yield spatio-temporal data. The odor of ripening fruit or the decay of a predator odor in a potential nest site yields information from the past (degraded odors), present (current strong odors) and future (the extrapolation of an odor to predict a future state). A cognitive agent responding to these odors (eating the fruit, rebuilding the nest) changes the landscape, which then changes the agent’s cognitive state. Thus, an agent’s responses to changes in the location or chemical composition of odors in its environment, whether sociochemical or other odors, fulfills the mutual manipulability criterion.

### Evolved cognition

If embodied cognition can be said to incorporate time scales of seconds to hours, then this raises the question of whether it can also include longer time scales, such as evolutionary time (Cheng 2018). For example, in niche construction, over evolutionary time, the actions of individuals alter their population’s environment, which then alters the value of future actions (Laland et al. 1999). Human culture is an extreme example of niche construction (Jablonka 2011), but it is a general principle of evolution; the foraging decisions of scatter hoarding squirrels results in the squirrels planting their own food trees (Robin and Jacobs 2022). Thus, niche construction should fulfill the mutual manipulability criterion. In addition, if processes such as niche construction show that cognition is indeed embodied over evolutionary time scales, then we should begin the analysis with the earliest forms of adaptive behavior, such as chemosensory behaviors.

Most animals rely to some extent on chemosensory modalities––to orient in space, find food, interact with conspecifics and avoid predators (Bargmann 2006). Using the metric of the size of gene families, animal species continue to invest more heavily in chemosensation than any other sensory modality: the largest gene families found in animals are those that encode olfactory receptors (Grus and Zhang 2008; Nei et al. 2008). Since the gene families are so large, it is possible to construct phylogenies for chemosensation dating back hundreds of millions of years, and to identify homologous olfactory receptor genes within the phylum Chordata.

Indeed, chemosensation is arguably the only cognitive trait that can be plausibly studied over the entire phylogenetic history and taxonomic breadth of animal species. For example, the Florida lancelet (*Amphioxus*), a basal chordate species, shares over 30 olfactory receptor genes with vertebrates (Niimura 2012) and similar reconstructions have been possible with the accessory or vomeronasal olfactory system (Grus and Zhang 2006, 2009).

Convergence in olfactory structure and function is another tool for the study of embodied cognition. Convergence has been found in the topography of neural circuitry, the structure of receptors and in the form of neural architecture, such as glomeruli, among the olfactory systems of molluscs, crustaceans, insects and vertebrates (Ache and Young 2005; Eisthen 2002). In insects and laboratory rodents, glomeruli allow for combinatorial encoding of odor mixtures. An odor object (e.g., ‘coffee’) is the perception of an object that is constructed from many, even hundreds, of monomolecular odorants (Wilson and Stevenson 2010). The odorants creating the percept of ‘coffee’, for example, can vary in numerous parameters which is why flavors can be so subtle, though the definition of a flavor includes input from the taste and somatosensory systems as well (Herz 2007; Shepherd 2013; Wilson and Stevenson 2010). The combinatorial nature of olfaction thus supports a massive capacity for encoding information (Kay et al. 2009; Laurent 2002).

The use of olfactory cognition by animals has clearly led to the construction of new ecological niches and shaped cognitive states over evolutionary time. Examples I have already mentioned include how orienting to odors may also have shaped the evolution of the vertebrate brain and the vertebrate hippocampus (Jacobs 2012; Jacobs and Menzel 2014). The tradeoffs between olfaction and respiration in lobe-finned fish that led to the first land vertebrates may also have shaped the evolution of the mammalian hippocampus and cognition. Air breathing led to the restriction to the olfactory system of encoding only air-borne odors. This could explain the increase in size and complexity of the vomeronasal system in terrestrial vertebrates. With the olfactory system becoming specialized for directional olfaction, the vomeronasal system could have taken over the role of the diagnostic olfaction of large, water-soluble signature mixtures, a role formerly performed by the olfactory system in their fish ancestors (Jacobs 2022).

Directional olfaction may also explain patterns of olfactory bulb size in paleontology, specifically the grade shifts in brain size in Jurassic mammals. Each increase in brain size during this geological period was preceded by an increase in the volume of the olfactory bulbs (Rowe et al. 2011). This paleontological record could be evidence for the evolution of increasingly complex spatial navigation to odors. As species increased their space use, this could have led to increases in trophic level and further abilities in spatial cognition (Jacobs 2012). This interpretation may also explain a result in a Miocene cercopithecoid primate. Here, too, the fossil record shows that large olfactory bulbs preceded an increase in sulci, a measure of cortical complexity (Gonzales et al. 2015). This could be further evidence that directional olfaction preceded an increase in behavioral complexity, which could have eventually bootstrapped the evolution of larger and more complex brains (Jacobs 2012).

A final example that underscores the importance of olfactory cognition in evolution comes from cetaceans, in particular the suborder of toothed whales (Odontoceti). With the exception of sea turtles, secondarily aquatic vertebrates have not regained the ability to smell odors dissolved in water (Kishida 2021), though shrews and moles smell odors underwater through air bubbles (Catania et al. 2008). Cetaceans (among other taxa, such as birds and catarrhine primates) have lost the second vertebrate olfactory system, the vomeronasal system (Meisami and Bhatnagar 1998). They also have reduced main olfactory systems. This loss of function is most extreme in the toothed whales; species in the suborder of baleen whales (Suborder Mysticeti) may detect air-borne odors while respiring and orient to odors associated with a local abundance of prey species (George et al. 2010). Yet, despite the sophisticated spatial orientation in three-dimensional space seen in toothed whales such as dolphins, porpoises and killer whales (Marino et al. 2007), the hippocampus is significantly smaller than expected for brain size in odontocetes (Patzke et al. 2013).

What is notable in this group is the simultaneous loss of the olfactory system and the reduction in hippocampus. The most parsimonious interpretation is that the hippocampus, a structure crucially involved in spatial orientation in terrestrial species (Jacobs and Schenk 2003), is indeed specialized for the use of odors in directional olfaction. The loss of directional olfaction would have been offset by the evolution of echolocation, an orienting mechanism suited to their new environment. Echolocation in toothed whales is convergent in function, even its genetic basis, with echolocation in bats (Order Chiroptera) (Jones and Teeling 2006; Teeling 2009). Directional olfaction in bats is constrained by their respiratory system, which may explain why echolocation also evolved in this group, which nonetheless retain diagnostic olfaction. All suborders of bats retain the main olfactory system and the hippocampus, and some species also retain the vomeronasal system (Bhatnagar and Meisami 1998). At present, the only explanation offered for this phylogenetic distribution of limbic structures, i.e., the hippocampus and the main olfactory system, is the olfactory spatial hypothesis (Jacobs 2022).

Because of the deep history and broad taxonomic breadth of chemosensory cognition, there are many parallels in insects and mammals in olfactory structure and function, as already discussed (Ache and Young 2005). The loss of directional olfaction in cetaceans may also have a parallel in insects, specifically in the secondarily aquatic water boatmen and water striders (Order Hemiptera). Just as cetaceans have successfully invaded the water with new sensorimotor adaptations, these insects have also replaced the use of olfactory signals. Instead, water striders communicate using ‘ripple signals’—tactile inputs from the seismic movements of the water surface generated by an individual or its conspecifics, i.e., mates and competitors (Han and Jablonski 2019; Wilcox 2016). In the insect brain, the multi-sensory associative structure is the mushroom body. Because of its major inputs from the olfactory input center (the antennal lobe), the mushroom body was long assumed to be a purely olfactory structure. This was challenged by data from water striders, which have a robust mushroom body but vestigial antennal lobes (Strausfeld et al. 2009). This is similar to the history of interpretations of the mammalian hippocampus. Before its role in spatial orientation was discovered (O’Keefe and Nadel 1978), the mammalian hippocampus was also considered to be only a ‘nose brain’ (rhinencephalon), for the same reason (Silveira-Moriyama et al. 2016).

Thus, there are parallels between echolocating cetaceans and the ripple-signaling water striders. Both groups have reduced or absent olfactory systems. Yet, in the dolphin, a multi-sensory associative center, e.g., the entorhinal cortex, is not reduced in size (Breathnach and Goldby 1954; Marino et al. 2007). Likewise in water striders, there is a loss of the olfactory structure, the antennal lobe, with no concomitant reduction in the mushroom body (Strausfeld et al. 2009).

The study of highly specialized ‘champion species’ have often led to the discovery of new principles of brain and behavior. As in the ‘cognitive fossil’ of nasal respiration modulating human memory (Jacobs 2022), the organization of cognition in dolphins and water striders might predict the organization of nervous systems in other taxa that have lost directional olfaction. This could shed light on questions about the function and evolution of multi-sensory associative centers (e.g., entorhinal cortex or mushroom body) and how they have changed, relative to the ancestral, olfactory state. Terrestrial animals invading the water––evolving into new bodies, with new actions creating new perceptions (e.g., echolocation and ripple signals) in a newly embedded and extended world––could lead to identifying first principles of embodied cognition.

## Conclusion

It is manifest that no theory of embodied cognition can be complete without including olfactory cognition, the common denominator of sensorimotor behaviors in living organisms. Yet, studies of cognition, by psychologists, philosophers and neuroscientists, have largely overlooked the importance of this sensory modality (Barwich 2019; Jacobs 2022; McGann 2017; Shepherd 2004).

The PROUST hypothesis seeks to redress this oversight by highlighting the embodiment of the sense of smell. Far from being an old and eccentric artifact of our evolutionary history, olfaction may represent the very scaffold of thought, the computation upon which complex brains evolved (Jacobs 2012). Recent advances in cognitive neuroscience, demonstrating the importance of nasal respiration for human memory, must put to rest any thought that this is a niche topic. Hence, to understand cognition, we must face the challenge of understanding this complex and understudied sensory modality, in particular directional olfaction (Jacobs 2012, 2022). By embracing our olfactory minds, perhaps a whole new PROUSTian world will appear, crossing time, space and evolutionary history.

## Data Availability

Data sharing not applicable to this article as no datasets were generated or analysed during the current study.
